# Development and validation of a psychosocial screening instrument for cancer

**DOI:** 10.1186/1477-7525-3-54

**Published:** 2005-09-07

**Authors:** Wolfgang Linden, Dahyun Yi, Maria Cristina Barroetavena, Regina MacKenzie, Richard Doll

**Affiliations:** 1 Psychology Department, The University of British Columbia, 2136 West Mall, Psychology/UBC, Vancouver BC, V6T 1Z4, Canada; 2 British Columbia Cancer Control Agency, Canada; 3 Health Care and Epidemiology, University of British Columbia, Canada

**Keywords:** Screening, distress, depression, anxiety, health-related quality of life, social support, norms, reliability, validity

## Abstract

**Background:**

We are reporting on the development of a psychosocial screening tool for cancer patients. The tool was to be brief, at a relatively low reading level, capture psychological variables relevant to distress and health-related quality-of-life in cancer patients, possess good reliability and validity, and be free of copyright protection.

**Method:**

Item derivation is described, data on reliability and validity as well as norms are reported for three samples of cancer patients (n = 1057; n = 570, n = 101).

**Results:**

The resulting 21-item psychological screen for cancer (PSCAN) assesses perceived social support, desired social support, health-related quality-of-life, anxiety and depression. It has good psychometrics including high internal consistency (alpha averaging .83, and acceptable test-retest stability over 2 months (averaging r = .64). Validity has been established for content, construct and concurrent validity.

**Conclusion:**

PSCAN is considered ready for use as a screening tool and also for following changes in patient distress throughout the cancer care trajectory. It is freely available to all interested non-profit users.

## Background

Cancer is now the leading cause of early death in Canada . A diagnosis of cancer is very emotionally threatening, may provoke anxiety or depression, and is difficult to live with because all aspects of life are overshadowed by the typical prognostic uncertainty [1-3]. Nevertheless, there is great variability in how patients respond to the diagnosis and this may be partly explained by the nature and quality of support that patients have, individual coping skills and by the meaning that they can learn to assign to this threat [4]. Psychological interventions for distress reduction can enhance quality-of-life, and help patients and families better cope [2,5,6] but distress often remains unrecognized and therefore untreated [7].

A number of critical questions for clinical practice arise from these insights, namely which intervention works best, and which patients are particularly needy and potentially responsive to a professional intervention. Screening for psychological distress and identification of emotional needs have important practical implications because practitioners want to responsibly serve their clientele and, given the scarcity of professional psychological resources, they want to make these resources available in the most equitable and efficient manner possible [5]. Researchers can help by extracting critical information from basic research for the explicit purpose of informing clinicians [8] and to ascertain best patient care.

Screening research has its own theoretical basis and concerns [1,5,9,10,12]. Screening can be expensive and has built into it the moral and professional imperative that one needs to act on urgent needs once identified. Along these lines, Rodin [8] and Ryan et al. [7] stress that screening and feedback does not necessarily lead to better patient outcomes unless such measures are accepted by the institutions and are supported by corresponding allocation of resources. However, only about one in four patients who report significant distress are actually referred to psychosocial care [9,13]. This, in turn, suggests that screening research is best done in a clinical setting with the active involvement of those professionals who are also key players in its intended subsequent routine implementation.

Given the high incidence of cancer, screening for distress requires tools that are psychometrically sound, inexpensive and quick, accepted by patients and staff, and of sufficient simplicity to be accessible to as many patients as possible [5]. In a review of the most frequently used tools for psychosocial distress screening [9], it became apparent that (a) most often measured are anxiety and depression, (b) there is no agreement on the best screening tool, (c) many of these measures are too long for routine screening, and (d) almost all of the tools used are copyrighted protected and would have to be purchased for every application. For the sake of parsimony, we decided to focus on psychological concepts that are know to be particularly critical for cancer patients, namely anxiety, distress [2,9], but also wanted to measure patient characteristics that reflect more positive aspects of life namely social support and quality-of-life [4,6,11,13]. Aside from the primary objective of developing a screening tool, we hoped that the psychometrics would support use of the same tool for tracking emotional adjustment in patients. Lastly, we wanted to include an instruction that questionnaire completion would imply permission for clinicians and researchers to directly contact patients to offer services or invite research participation. If agencies and patients give such permission, both clinical service provision and patient identification for research are made much less cumbersome.

In light of these observations, we intended to develop a tool that embraced all of the desired features listed above. While our work on a brief psychosocial tool does not claim to break completely new ground (see the review of previously used screening tools [9] and the NCCN guidelines [5], we posit that it stands out because of (a) its brevity, (b) its development in the clinical context where it was to become implemented, (c) the scope of the domains included, (d) that it measures both negative and positive aspects of the patients' quality of life, and (e) its non-commercial nature. A series of three studies (subdivided into Phase Ia, Ib, and Phase II) was planned to establish psychometrics and norms in two test phases. The objectives for Phase Ia were determination of item clarity, basic reliability, and validity features including internal consistency, desired and undesired factor correlations as a test of construct validity, as well as to generate initial norms for a cancer patient population. A second sample was tested in Phase Ib to also identify cancer-type specific norms, and gender-specific norms.

For Phase II, a third (smaller) sample was evaluated initially and then retested for establishment of test-retest reliability; in addition, participants completed a larger test package that permitted concurrent validation of the PSCAN subscales with well established and substantially longer versions of tests that we considered "industry standard" in order to show adequacy of content sampling and concurrent validity for PSCAN.

## Methods

### Phase I

#### The instrument

After extensive discussion of existing instruments and their respective strengths and weaknesses, the authors and a group of practicing clinicians agreed to focus on anxiety, depression, social support, and health-related quality-of-life. The final version of the full scale as described here is found in the Additional file 1 as are instructions for how to obtain permission for its use from the authors. (Given that no acceptable measure of anxiety and depression could be found that did not have to be purchased from a commercial publisher, a number of popular scales were studied to obtain a clear sense of content domains). Five items each were written to elicit patients' level of anxiety and depression. Each item is scaled as 1–5 ('not at all' to 'very much so')

The social support items are derived from a social support scale used in the Epidemiological Study of the Elderly [14] that is considered to be in the public domain. It provides 5 items that, when clustered, are referred to as Social Network and Support Assessment (SNSA) which taps into available informational, instrumental, and emotional support. One additional item (not part of the SNSA) asks how much social support people desire (Item 6). The SNSA has been reported to have internal consistencies of .47 to .61 [15]. The SNSA was found to predict mortality in epidemiological studies [14] and has also been used in cancer populations [15]; the existence of distinct subscales was shown to possess discriminant validity because therapy-induced changes were apparent on instrumental and informational support but not on other items [15].

Given that desired support and received support generally do not intercorrelate and that mismatched support attempts are not constructive [6,16], the 'desired support' item was written as a single item scale with a 1–9 rating ('not at all' to 'very much so') to permit variability in ratings. This single item scale has already shown remarkable clinical usefulness because Krumholz et al. [17] found that, in a sample of 292 elderly patients with heart failure, those patients *not seeking and not receiving support *were three times more likely to be alive one year later that those patients *who did seek support but did not receive it*. Apparently, social support is only useful when its availability is actually also desired. All other social support items (items 1–5) are rated as yes/no and coded 0 or 1. While the direction of scoring is ultimately arbitrary, we treated positive support ratings as high scores.

Quality-of-life measures are generally distinguished as being either broad and generic or disease-specific with researchers favoring the inherent sharper focus of disease-specific tools. That notwithstanding, we did not decide on a highly disease-specific measure because many distressing physical aspects of cancer (like pain and functional limitations) are only salient in late stage cancer and are, fortunately, of limited importance to the lives of early-stage cancer patients who form the majority of study participants. We therefore decided to keep the assessment of health-related quality-of-life (HRQoL) sufficiently broad and generic to embrace cancer patients in all stages. The chosen items are from the Health Related Quality of Life questionnaire developed by the Centers for Disease Control [18] and are part of the Behavioural Risk Factor Surveillance System since 1993. The questions seek to learn about HRQoL by distinguishing between global, self-rated health and numbers of days negatively affected by poor mental or physical health (PSCAN items 7–11). Test-retest stability of this tool was established in a sample of 868 adults with kappas ranging from = .58 to .75; reliability was not affected by gender or race [19]. The scale has also been used in a Canadian sample of 926 men and women age 65 or over and revealed that those with poor self-rated health showed a 17-fold increase in the number of unhealthy days [20].

### Study population

#### Sample 1

Participants were 1057 consecutive cancer patients coming into first contact with the Vancouver Cancer Centre (i.e., after a positive diagnosis had been established). No demographic information was collected at this time because the data were only to be used in the item development process and not any kind of hypothesis testing. Participants were asked by the receptionist to complete the 21-item PSCAN after they had read the instructions and agreed to participate. The plan was to collect initial data for a 3-month time period rather than setting a particular sample size as a target; the rationale for this decision was to allow us later determination what percentage of the total number of eligible patients had actually participated; of all patients who had made initial contact with the cancer agency during this same time period, about 90% had indeed completed PSCAN which suggests that this sample is quite representative of the typical patient population seen by this agency.

Feedback from patients suggested that the wording of two items was somewhat ambiguous and these were subsequently changed prior to the recruitment of sample 2.

#### Sample 2

Participants were 547 consecutive cancer patients (average age 66.5 yrs (SD = 14.5), 304 women and 243 men) coming into first contact with the Fraser Valley Cancer Centre (i.e., after a positive diagnosis had been established). The procedure was the same as the one applied to sample 1.

### Phase II

The sample consisted of 101 cancer patients making first contact with the BC Cancer Agency at the Vancouver Center (41 male, 60 female). Eligible patients were recruited consecutively by two trained research assistants over a period of one month. The research assistants were physically located in the reception area, were alerted about potentially eligible patients by the receptionist, and then approached patients individually to explain the study, seek consent, and request completion of a test package. Patients were explicitly recruited to participate in a test-retest study and indicated whether they preferred recontact by mail or telephone. Two months later, patients were recontacted according to their preferred method. If no contact could be made by telephone after three attempts, no further attempts were made. Patients who received the questionnaire package by mail, were not further reminded to return them. This relatively 'low pressure approach' still resulted in a 65% return rate of completed retest materials. The questionnaire package consisted of the PSCAN as described above, the Hospital Anxiety and Distress Scale (HADS) and the ENRCHD Social Support Instrument [ESSI; [21,22]].

The HADS is a very frequently used 14-item scale tapping anxiety and depression. Bjelland et al. [23] provided a review of the psychometrics of HADS based on 747 published studies and reported Cronbach's alphas of .68 to .93 for anxiety and .67 to .90 for depression. Factor analyses routinely confirm the underlying 2-factor structure.

The ESSI is a 7-item instrument with strong test-retest reliability (means one month apart were 27.8 and 27.8), and internal consistency in a sample of 271 cardiac patients was .88. Concurrent validity was shown by relating ESSI scores to established psychiatric diagnoses of depression and an index of social functioning [21,22].

## Results

The findings obtained from the three samples during Phase I and II testing are presented in aggregated form that presents findings organized around the test's properties regarding means and reliability (Tables 1, 2, 3), and then reports on evidence of validity. Finally, raw means and standard deviations are provided for each cancer type and each gender group (Table 4). This presentational approach appeared more parsimonious and provided a more logical organization than a mere sequential listing of each temporal step of the result finding process.

**Table 1:** Subscale Descriptive Data (Means, and variability), Phase I

	Range of scores	Mean sample 1	SD sample 1	Mean sample 2	SD sample 2
SS_Total	0–5	4.6	.82	4.5	.99
SS_Desired	0–10	4.0	3.5	3.7	3.4
QOL, Perceived	0–20	6.5	5.4	6.7	5.5
QOL, Days	0–120	31.6	29.3	24.0	24.3
Anxiety	5–24	8.1	3.8	8.2	4.2
Depression	5–25	8.1	3.9	8.2	5.1

**Table 2:** Internal Consistency

**Internal Consistency**	**Alpha Sample 1**
QOL_Days	0.79
QOL_Perceived	0.89
Anxiety	0.83
Depression	0.79

**Table 3:** Test-retest stability

**Subscale**	**Stability coefficient r**	**M and SD Time 1**	**M and SD Time 2**
Social Support Total	.87	5.3 (.73)	5.3 (.74
Social support desired	.59	4.2 (3.3)	4.4 (3.4)
Anxiety	.67	7.4 (3.3)	6.6 (2.4)
Depression	.61	7.7 (3.8)	7.0 (2.4)
QoL Perceived	.59	4.2 (4.3)	5.6 (5.6)
QoL Days	.49	22.3 (26.5)	20.5 (27.4)

**Table 4:** Subscale means for each gender and cancer type

**Cancer Type**	**Subscale – Mean (SD)**					
	Depression	Anxiety	QoL-P	QoL-D	SS-Tot	SS-Des
**Gastro-Intestinal**						
Female (n = 39)	18.3 (12.9)	8.4 (3.7)	6.2 (5.7)	26.7 (23.9)	4.9 (.5)	4.0 (3.9)
Male (n = 39)	13.3 (4.4)	6.7 (2.7)	7.1 (4.6)	31.3 (21.7)	4.9 (.4)	2.7 (3.5)
**Lung**						
Female (n = 35)	17.6 (8.6)	9.2 (4.5)	8.7 (4.9)	30.6 (22.9)	4.0 (1.2)	4.4 (3.6)
Male (n = 42)	17.0 (6.9)	8.9 (5.6)	11.7 (6.1)	41.3 (22.8)	4.6 (.7)	3.9 (3.2)
**Lymphoma**						
Female (n = 12)	22.6 (8.6)	12.1 (4.4)	9.9 (7.9)	35.6 (28.4)	4.9 (.3)	5.9 (4.0)
Male (n = 13)	19.6 (11.9)	9.6 (6.4)	11.0 (6.6)	35.0 (24.6)	4.7 (.5)	4.2 (3.9)
**Breast**						
Female (n = 133)	15.6 (6.5)	7.9 (4.3)	5.0 (4.4)	21.3 (21.3)	4.5(.9)	3.8(3.4)
Male (n = 1)	10.0 (na)	5.0 (na)	2.0 (na)	0 (na)	5.0 (na)	5.0 (na)
**Melanoma**						
Female (n = 9)	14.5 (7.1)	8.3 (5.1)	2.1 (3.4)	12.3 (21.5)	4.7 (.5)	5.0 (3.5)
Male (n = 12)	13.2 (4.2)	6.6 (1.8)	3.9 (2.6)	4.2 (5.2)	4.6 (.7)	1.7 (2.2)
**Head & Neck**						
Female (n = 12)	26.1 (16.0)	11.0 (3.7)	8.7 (5.9)	25.0 (21.9)	4.3 (.8)	5.6 (3.2)
Male (n = 18)	15.3 (5.8)	8.3 (4.0)	6.6 (5.8)	20.8 (24.7)	4.5 (1.2)	3.6 (3.1)
**Genito-Urinary **(incl Prostate)						
Female (n = 5)	12.0 (2.8)	6.2 (2.2)	6.4 (3.8)	17.4 (24.8)	4.8 (.5)	1.3 (1.5)
Male (n = 83)	14.6 (6.5)	7.4 (3.5)	5.9 (5.1)	14.6 (27.6)	4.4 (1.2)	2.8 (3.4)

QoL-P = Quality-of-Life Perceived; QoL-D = Quality-of-Life Days; SS-Tot = Social Support Total; SS-Des = Social Support Desired

As the mean scores in Table 1 show, indices of variability reveal that participants used a wide range of scores that in turn suggests that PSCAN is sensitive in discriminating among patients. Means for the two samples were quite similar.

### Reliability

As Table 2 reveals the internal consistencies for these four subscales are high and satisfy traditional cutoffs for full-length tests despite their brevity in PSCAN. Internal consistency was not computed for the Social Support items, because each item was designed to tap somewhat different dimensions of support and the yes/no scoring method did not create much item response variability that could then be meaningfully analyzed.

Scores for social support variables were very stable over 2 months whereas QOL and distress-related variables showed less stability although they were still moderately high (Table 3).

### Validity

A number of steps were undertaken to establish construct validity. Basic requirements for test creation [24] are stated below and corresponding results are listed for each:

(a) the items that make up a distinct subscale (and that presumably reflect an underlying 'factor') intercorrelate with each other (but not so highly that they suggest duplication) and that they load (i.e., correlate) with the total subscale score. Given the complexity of results, they are not reported in detail but the pattern of results clearly indicates that this requirement was met. For example, the Quality-of-Life items correlated between r = .49 and r = .95 with the average of inter-item correlations being r = .73.

(b) the subscale scores themselves should correlate with each other if they are conceptually related. Based on extant literature, it is expected that anxiety and depression will partly overlap, and high social support and QoL should correlate to some degree with low depression and anxiety. This was confirmed with r's ranging from .55 to .92.

(c) generally, subscale scores across domains should not highly intercorrelate with each other because that would suggest redundancy that is especially undesirable in a brief screening tool. These test properties were determined with a series of correlational analyses and supported the notion of minimal overlap in general [20]. The data suggest that the three 'QoL days' items and the two 'QoL Perceived' items overlap but still tap different aspects of QoL; each respective item correlates highly with its own subscale total score. This finding supports the continued inclusion of these two sets of QoL items.

Anxiety and depression were predictably intercorrelated (r = .71 and r = .55 in sample 1 and 2 respectively) and explain about 30–50% of each other's variance.

Correlation coefficients of the rather short PSCAN subscales with equivalent longer version from established tests strongly support concurrent validity. The r-scores for samples 1 and 2 respectively were .72 and .82 for anxiety; .59 and .75 for depression, and .61 and .61.

Lastly, we computed means for men and women and each subscale as a function of cancer type. Only those types of cancer were listed where at least 5 men and 5 women were found to fill each cell (with the exception of frequent cancers that are only found in one gender). Results are displayed in Table 4. No inferential testing was conducted because the power for tests varies greatly as a function of the varying sample sizes per cell; we do, however, report effect sizes because this display of psychological profiles for all cancer types can serve as a hypothesis generator for future studies and may enable power calculations for sample size estimation.

It is tempting to interpret the results shown in Table 4 but given that no inferential tests were computed, any interpretation is speculative should be kept at a minimum. It appears that men typically report less negative affect than women and that there is considerable variability in distress and QoL as a function of cancer type. This suggests an urgent need for the accrual of a larger sample including all cancer types such that sufficiently powered inferential tests can be conducted.

## Discussion

The objective of this tool development process was to gather enough information so that readers could potentially make a decision to adopt the scale for their own use with cancer populations, knowing that adequate reliability and validity testing had been undertaken. We consider the psychometric characteristics of PSCAN to be satisfactory especially when considering that it is a very brief tool [6]; the scale characteristics typically met even the desired standards for longer scales. Subscale means for two large samples recruited at different sites were very similar suggesting stability of the scale scores. Practitioners can now choose to use PSCAN instead of the recommended single-item distress thermometer [5] or use it in a complementary fashion, as a second step, if the single item distress thermometer suggests elevated distress. A second advantage of PSCAN is that the standard instructions can include a statement about the patient having consented to be contacted for offers of professional help or participation in research. This feature has been found very useful in clinical settings where research is also being conducted because Ethics Reviews Boards do not usually permit direct contacting (i.e., "cold calls") of patients for study recruiting.

### Reliability

Internal consistency was good across the two independent samples and test-retest stability was also acceptable. Note that at the time of recruitment for the test-retest study (Phase II), patients had come for their first visit to the cancer center. During the 8-week interval for test and retest, these patients typically learned more about their diagnosis and many began active treatment. It was therefore expected that these experiences would affect distress and quality-of-life, and that the test-retest scores would only be moderately high. In fact, had the test-retest scores come close to r = 1, this would have suggested that PSCAN was insensitive to measuring change and that would have been indicative of a significant weakness in the test.

### Validity

Individual item correlations with their respective subscale scores were high, suggesting that they load appropriately on the constructs to be measured. All relationships between constructs measured by the PSCAN were of a strength and direction so as to be consistent with the literature and that, in turn, suggests that PSCAN possesses construct validity. The newly written anxiety and depression items not only formed cohesive subscales with some predicted overlap of anxiety and depression but also correlated highly with longer versions of anxiety and depression scales of well established tools thus indicating high concurrent validity. There is no firm consensus on how to establish content validity other than finding that experts agree. The research group who developed PSCAN, the cancer agency staff who worked with it, and the patients who responded, all considered the items as meaningful and sensitive. It was also interesting to see that once Phase I had been completed and PSCAN-derived patient distress information became known to the psychosocial support staff, they reported quickly occurring changes in their clientele's composition because the patients now seen by family and patient counseling included more men and more minority patients who apparently had gone undetected by previous practice patterns. Interestingly, the use of PSCAN and especially the inclusion of an item on suicidal thinking, also led the agency' s staff to review and clarify their policy on how to respond to highly distressed, potentially suicidal patients. Hence, the development of this screening tool by a 'mixed' team of researchers and service providers also led to ready acceptance of the tool by service providers and triggered prompt changes in their practice patterns. This could be considered a form of ecological validity.

## Conclusion

We conclude that there is sufficient psychometric evidence to support PSCAN's use as a screening tool and possibly as a tool for tracking patient changes in emotional well-being. Consistent with the larger literature on distress screening, it is likely that PSCAN's strength is in sensitivity of detection, not in specificity; in order to advance to a full-fledged psychiatric diagnosis, further standardized testing as well as individual histories and assessments will need to be considered. At this time, there are enough normative data available so that one can decide on a clear, replicable cutoff based on percentile scores such that an agency might decide to offer psychological services to all patients scoring above 80^th ^percentile of depression for example. However, we believe strongly that more information is needed before establishing a cutoff that also has a clinical meaning, like a demonstration of the health consequences of not offering services to patients above a given cutoff. We also recommend that norms be compiled for a large, representative cancer sample that fairly represent both sexes, all cancer subtype populations, and cultural minorities. Compilation of norms for different stages in the trajectory of cancer care should equally be considered. This, in turn, will aid empirically-based, future decisions about use of cutoffs [25]. Furthermore, it will be important to collect normative data from a physically and psychologically healthy sample and another medical sample so as to place the data obtained from cancer patients in a larger population context, and to serve as reference levels. Although PSCAN was explicitly developed and validated for cancer patients, the measured concepts are not uniquely relevant for cancer but appear pertinent for other chronic disease populations as well [1].

Lastly, PSCAN, is freely available to non-profit users who need, however, to contact the copyright holder for permission and conditions of use via our website: .

Instructions for scoring and status quo of knowledge about norms and cutoffs will be made available at that time.

## List of abbreviations

ESSI ENRICHD Social Support Instrument

HADS Hospital anxiety and Distress Scale

PSCAN Psychosocial Screen for Cancer

HRQoL Health-Related Quality of Life

SNSA Social Network and Support Assessment

## Supplementary Material

Additional File 1 Linden additional file.doc **PSCAN – Psychological Screening Tool.** Click here for file
