# Supplementary material for: Development and validation of a psychosocial screening instrument for cancer
Source: Health Qual Life Outcomes. 2005 Sep 7;3:54. doi: 10.1186/1477-7525-3-54 (PMC1215503; doi:10.1186/1477-7525-3-54)
Supplement: Additional File 1 — Linden additional file.doc PSCAN – Psychological Screening Tool. [file 1477-7525-3-54-S1.doc]

# PSCAN – Psychological Screening Tool

***Please answer the following questions to help us learn more about your well being. A serious illness can affect the quality of your life in many ways. We may contact you to offer our counseling services based on the information you provide to us, or contact you regarding opportunities to participate in research.***

***Part A* Please respond to each question with a simple "Yes" or "No" by making a circle around the appropriate answer or by circling a number. There are no right and wrong answers.**

**1. Do you live alone? YES NO**

**2. When you need help, can you count on YES NO**

**anyone to help with daily tasks like grocery**

**shopping, cooking, giving you a ride?**

**3. Do you have regular contact with friends YES NO**

**or relatives?**

**4. Have you lost your life partner within the YES NO**

**last few years ?**

**5. Can you count on anyone to provide you YES NO**

**with emotional support?**

**6. Do you feel that you want and need this kind of emotional support ?**

**No, not at all 0 1 2 3 4 5 6 7 8 9 10 Very much**

# Part B: Please circle the number that best describes how you feel:

**7. Would you say that in general your health is**

**Very Poor 0 1 2 3 4 5 6 7 8 9 10 Excellent**

**8. Would you say that in general your quality of life is**

**Very Poor 0 1 2 3 4 5 6 7 8 9 10 Excellent**

**9. Now thinking about your physical health, which includes physical illness and injury, for how many days during the past 30 days was your physical health not good? _________days**

**10. Now thinking about level of stress, depression, and problems with emotions, for how many days during the past 30 days was your mood not good? __________days**

**11. During the past 30 days, for about how many days did poor physical or emotional health keep you from doing your usual activities, such as self-care, work, or recreation? _________days**

***Part C:* *Please place an ‘x’ in the box that best describes what you have experienced***

|  | Not at  all | A little  bit | Moderately  so | Quite  a bit | Very much  so |
| --- | --- | --- | --- | --- | --- |
| 12. ***During the past week*** I have felt that my  heart races and I tremble. |  |  |  |  |  |
| 13. ***During the past week*** I have felt that I  cannot control anything. |  |  |  |  |  |
| 14. ***During the past week*** I have lost interest in  things I usually cared for or enjoyed. |  |  |  |  |  |
| 15. ***During the past week*** I have felt nervous  and shaky inside. |  |  |  |  |  |
| 16. ***During the past week*** I have felt tense and  can’t relax. |  |  |  |  |  |
| 17. ***During the past week*** my thoughts are  repetitive and full of scary things. |  |  |  |  |  |
| 18. ***During the past week*** I have felt restless  and find it difficult to sit still. |  |  |  |  |  |
| 19. I have ***recently*** thought about taking my life. |  |  |  |  |  |
| 20. ***In the past year***, I have had 2 weeks or more  during which I felt sad, blue, or depressed. |  |  |  |  |  |
| 21. ***I have had 2 years or more in my life*** when  I felt depressed or sad most days even if  I felt o.k. sometimes. |  |  |  |  |  |

**Thank you for taking the time to respond to this form.**
